# Bacterial endophyte communities of three agricultural important grass species differ in their response towards management regimes

**DOI:** 10.1038/srep40914

**Published:** 2017-01-19

**Authors:** Franziska Wemheuer, Kristin Kaiser, Petr Karlovsky, Rolf Daniel, Stefan Vidal, Bernd Wemheuer

**Affiliations:** 1 Section of Agricultural Entomology, Department of Crop Sciences, Georg-August-University Göttingen, Grisebachstr. 6, D-37077 Göttingen, Germany; 2 Department of Genomic and Applied Microbiology and Göttingen Genomics Laboratory, Institute of Microbiology and Genetics, Georg-August-University Göttingen, Grisebachstr. 8, D-37077 Göttingen, Germany; 3 Molecular Phytopathology and Mycotoxin Research, Department of Crop Sciences, Georg-August-University Göttingen, Grisebachstr. 6, D-37077 Göttingen, Germany

## Abstract

Endophytic bacteria are critical for plant growth and health. However, compositional and functional responses of bacterial endophyte communities towards agricultural practices are still poorly understood. Hence, we analyzed the influence of fertilizer application and mowing frequency on bacterial endophytes in three agriculturally important grass species. For this purpose, we examined bacterial endophytic communities in aerial plant parts of *Dactylis glomerata* L., *Festuca rubra* L., and *Lolium perenne* L. by pyrotag sequencing of bacterial 16S rRNA genes over two consecutive years. Although management regimes influenced endophyte communities, observed responses were grass species-specific. This might be attributed to several bacteria specifically associated with a single grass species. We further predicted functional profiles from obtained 16S rRNA data. These profiles revealed that predicted abundances of genes involved in plant growth promotion or nitrogen metabolism differed between grass species and between management regimes. Moreover, structural and functional community patterns showed no correlation to each other indicating that plant species-specific selection of endophytes is driven by functional rather than phylogenetic traits. The unique combination of 16S rRNA data and functional profiles provided a holistic picture of compositional and functional responses of bacterial endophytes in agricultural relevant grass species towards management practices.

Endophytic bacteria comprising various genera have been detected in a wide range of plant species^1^. Beneficial endophytic bacteria can promote plant growth and/or resistance to diseases and environmental stresses by a variety of mechanisms. These include the fixation of atmospheric nitrogen^2^,^3^ or the production of antibiotics and phytohormones^4^,^5^,^6^. As consequence, several endophytes are used in agricultural cropping systems as biofertilizers and/or biological control agents^4^,^5^. Due to their ubiquitous occurrence in plants as well as their ecological and economic relevance, it is important to decipher the interactions between bacterial endophytes, their host plants, and agricultural practices.

Recent research has shown that bacterial endophytic communities in plants within the *Poaceae* family are affected by fertilizer application^7^,^8^,^9^. In two previous studies, fertilizer application influenced the nitrogen-fixing (diazotrophic) bacterial community in maize roots and stems^8^ as well as in rice roots^10^. The impact of agricultural practices on the entire bacterial community in aerial plant parts of grass species has been rarely investigated as most previous studies focused on the effect of fertilizer application on diazotrophic and/or root endophytic bacteria in a single grass species^8^,^10^,^11^,^12^,^13^,^14^. Moreover, comparative studies on structural and functional responses of bacterial endophyte communities colonizing different grass species towards agricultural practices are missing.

In the present study, we investigated the influence of different management regimes on bacterial endophyte communities in the agriculturally important grass species *Dactylis glomerata* L., *Festuca rubra* L., and *Lolium perenne* L. We selected these grass species because they differ in their grassland utilization indicator values^15^. These values provide information on the tolerance of plant species towards management such as mowing or grazing^16^. Both *D. glomerata* and *L. perenne* have a higher tolerance against mowing compared to *F. rubra* while *L. perenne* shows a higher indicator value for nitrogen than the other two grass species^15^. Plant samples were taken from the GrassMan experimental field^17^. This unique experimental site was established to study biodiversity along an agricultural management intensity gradient. Previously, we analyzed the effect of fertilizer application and mowing frequency on bacterial endophyte communities in the three grass species by denaturing gradient gel electrophoresis (DGGE)^9^. The DGGE study suggested that mowing frequencies in combination with fertilizer application had a significant impact on endophyte community structures and that this effect was grass species-specific. Although this approach allowed a quick and efficient method to examine structural changes of bacterial endophyte communities, the phylogenetic resolution of this approach was limited.

In the current study, using the same biological material as in our previous work^9^, we sequenced the bacterial 16S rRNA gene to gain deeper insights into composition and compositional changes of bacterial endophyte communities in response to fertilizer application and mowing frequency. To better understand plant-microbe interactions with respect to management regimes, correlation-based indicator species analyses were performed^18^. In addition, functional profiles (artificial metagenomes) were calculated from obtained 16S rRNA gene data using Tax4Fun^19^ to investigate functional responses of endophyte communities to applied management regimes. This is of particular importance as differences in community function between various grass species and the functional responses towards management regimes have not been addressed so far. We focused on three main hypotheses: (i) bacterial endophyte diversity and community composition differ among the investigated grass species; (ii) endophyte diversity and community composition respond in a grass species-specific manner to the different management regimes; and (iii) bacterial functioning is altered in a similar manner as bacterial endophyte community composition and diversity. The results contribute to the understanding how different management regimes affect bacterial endophyte diversity, community composition, and endophyte functioning in agriculturally important grass species.

## Results

### Overall bacterial endophyte community

We collected aerial plant parts of the agriculturally important grass species *Dactylis glomerata, Festuca rubra*, and *Lolium perenne* over two consecutive years (Supplementary Material Table S1). In total, 71 plant samples were taken from the GrassMan Experimental Field (Supplementary Material Fig. S1). The response of bacterial endophyte communities of these grass species towards different management regimes was assessed by pyrotag sequencing of 16S rRNA genes. After quality filtering, denoising, and removal of potential chimeras as well as non-bacterial sequences, more than 48,000 high-quality sequences were obtained for further analysis. Sequences clustered into 1,076 bacterial OTUs at 97% genetic identity (Supplementary Material Table S2). Rarefaction curves (see Supplementary Material Fig. S2) as well as calculated diversity indices (Supplementary Material Table S3) revealed that approximately 50% of the bacterial diversity (number of OTUs) was recovered by the surveying effort. All OTUs were classified below phylum level.

**Figure 1:**
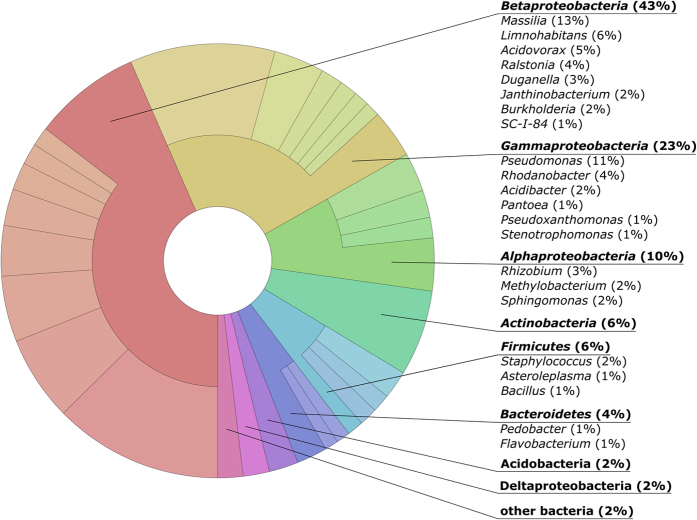
Abundant bacterial phyla and genera in the three investigated grass species. Only groups with an average abundance >1% in at least one of the investigated grass species are shown.

Five dominant phyla (>1% of all sequences across all samples) were present in each sample and accounted for more than 97% of all sequences analyzed (Fig. 1). *Proteobacteria* were predominant across all samples (79.2%). Other abundant phyla were *Actinobacteria* (6.4%), *Firmicutes* (6%), *Bacteroidetes* (4.3%), and *Acidobacteria* (1.9%). At genus level, *Massilia* (12.6%) was predominant across all samples (Fig. 1). Other abundant genera observed in this study were *Pseudomonas* (10.8%), *Limnohabitans* (6.4%), *Acidovorax* (4.9%), *Rhodanobacter* (3.8%), *Ralstonia* (3.8%), *Rhizobium* (2.9%), *Janthinobacterium* (2.1%), and *Bacillus* (1.4%).

### Bacterial endophyte community composition is strongly influenced by grass species and management regimes

**Figure 2:**
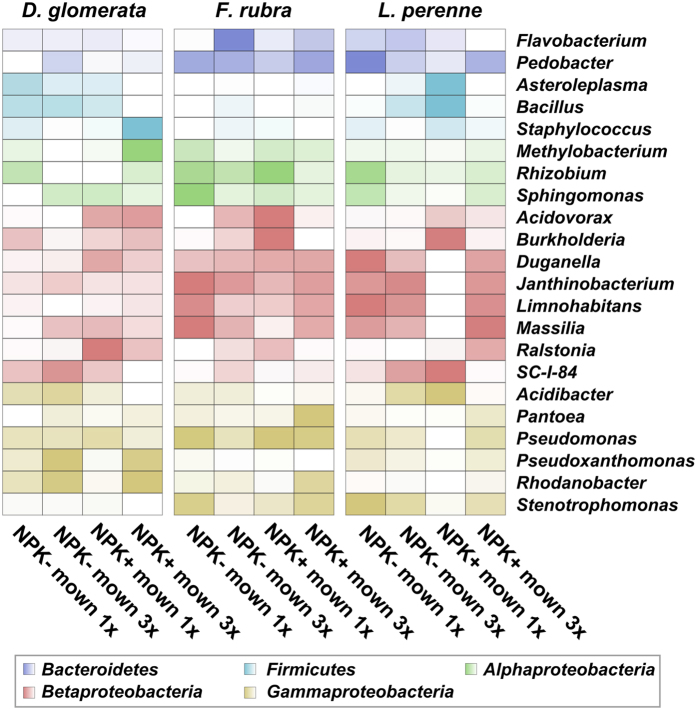
Abundance of prominent bacterial genera in the three investigated grass species. Only groups with an average abundance >1% in the entire dataset are shown. The color code refers to sequence abundance, with high abundances (dark colors) and low abundances (bright colors). Abbreviations: no fertilizer application, NPK-; with fertilizer application, NPK+; mown three times a year, 3x; mown once a year, 1x.

According to our first hypothesis that bacterial endophyte diversity and community composition would differ between the three grass species, we compared bacterial diversity (represented by the Shannon index H’) and richness (number of observed OTUs) with regard to the three grass species (Table 1). Bacterial diversity was significantly affected by grass species in both sampling years whereas richness was not affected. In addition to differences in diversity, grass species significantly influenced endophytic community composition in both sampling years (Table 2). Plant species explained 12.92% and 12.1% (Bray-Curtis distances) or 17.88% and 10.11% (Weighted UniFrac distances) of the variance in the dataset in 2010 and 2011, respectively. Several genera were more abundant in one or two of the investigated grass species. Higher abundances were recorded for several genera including *Duganella, Janthinobacteirum, Limnohabitans, Massilia, Pedobacter, Flavobacterium*, and *Stenothrophomonas* in *F. rubra* and *L. perenne* compared to *D. glomerata* whereas the opposite was found for *Pseudoxanthomonas* and *Rhodanobacter* (Fig. 2).

We further expected that endophyte diversity and community composition would respond in a grass species-specific manner to the different management regimes. We did not observe any direct influence of fertilizer application and mowing frequency on bacterial richness and diversity in both sampling years (Table 1). Nevertheless, fertilizer application had a marginally significant impact on bacterial endophyte richness in the three grass species investigated in 2010. In addition, the combination of plant species with fertilizer application or with mowing frequency significantly affected bacterial endophyte diversity both in 2010 and 2011. The influence of management regimes on bacterial community profiles was analyzed by permutational multivariate analysis of variance (PERMANOVA) (Table 2). A marginally significant effect of fertilizer application was recorded in 2011 but not in 2010 when using Bray-Curtis distances. The interaction of plant species and fertilization regime explained more than 21% of the variance in both sampling years when using Bray-Curtis distances. The coefficient of determination was higher in 2010 while the interaction of plant species and fertilization regime had only a marginally significant effect on endophyte community structures in 2011 when employing weighted UniFrac distances. Although mowing frequencies (once vs. thrice a year) alone did not exhibit any significant impact on community structures in both sampling years, more than 41% of the variance in the dataset was explained by a combination of plant species, fertilizer application, and mowing frequencies (Table 2).

Furthermore, we analyzed the effect of management regimes on bacterial endophytes in each grass species. The combination of fertilizer application and mowing frequency significantly influenced bacterial endophyte richness in *D. glomerata* in 2010 while only a marginally significant effect on endophyte richness in *D. glomerata* in 2011 and in *F. rubra* in both sampling years was observed (Table 1). Moreover, fertilizer application changed significantly the community structure in *D. glomerata* in 2010 but not in 2011 (Table 2). The combination of fertilizer application with mowing frequencies significantly affected bacterial community composition in *D. glomerata* in 2010, explaining 37.65% and 41.13% of the variance when employing Bray-Curtis and weighted UniFrac distances, respectively (Table 2). In addition, the combination of fertilizer applications with mowing frequencies significantly influenced affected bacterial community composition in *L. perenne* (Bray-Curtis distances) in 2010, but only marginally in 2011 (weighted UniFrac distances), explaining 34.55% and 44.47% of the variance, respectively. Neither mowing frequencies nor fertilizer application exhibited any significant influence on community structures in *F. rubra* in both sampling years.

The abundances of bacterial genera differed not only among the three grass species, but also between the four management intensity levels (Fig. 2). Four general trends were observed (1) decrease in abundance with increasing management intensity (e.g., *Bacillus, Acidibacter*, and *Asteroleplasma* in *D. glomerata; Acidibacter* in *F. rubra*), (2) increase in abundance with increasing intensity (e.g., *Pantoea* and *Rhodanobacter* in *F. rubra; Ralstonia* in *L. perenne*), (3) roughly constant abundances across all four management regimes (*Stenotrophomonas* and *Pantoea* in *D. glomerata; Rhonadobacter* and *Methylobacterium* in *L. perenne*), and (4) no distinct patterns along management intensity (e.g., *Acidovorax, Rhizobium*, and *Pseudomonas* in *F. rubra* and/or *L. perenne*). These results were not consistent between the three grass species. For example, the abundance of the genus *Stenotrophomona*s in the endosphere of *D. glomerata* was not influenced by the management regimes while the opposite was observed for *F. rubra* or *L. perenne*. In addition, *Methylobacterium* and *Staphylococcus* had higher abundances in *D. glomerata* grown on plots with high management intensity (fertilized and mown three times a year) while this effect was not observed in the other two grass species. Mowing frequency altered the abundances of several bacterial genera. For example, we recorded higher abundances of *Flavobacterium* in *F. rubra* as well as of *Pseudoxanthomonas* and *Rhodanobacter* in *D. glomerata* grown on plots mown three times a year.

### Bacterial taxa associated with grass species and fertilizer application

**Figure 3:**
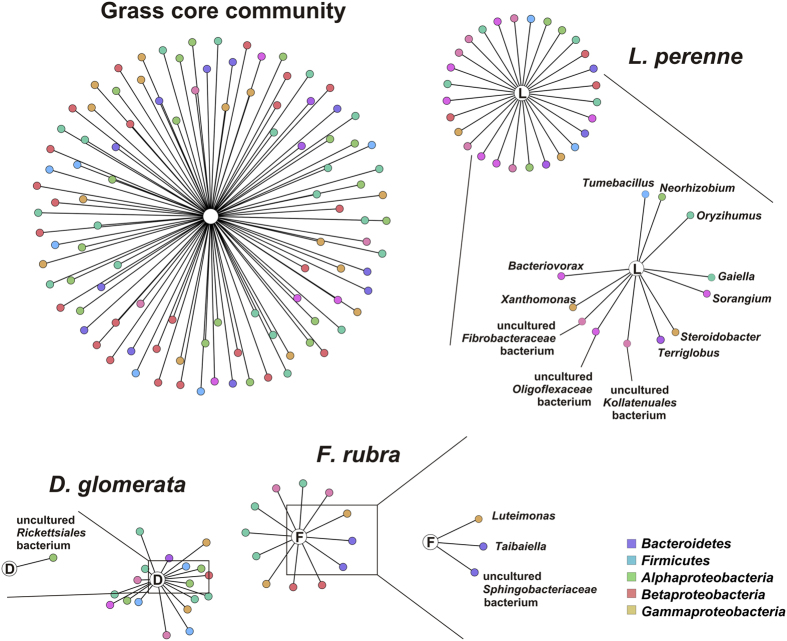
Associated bacterial taxa (at genus level). Only uniquely associated taxa or those associated with all three grass species (designated as grass core community) are shown. Circular layouts represent all associated taxa while significant associations are enlarged for each grass species. Abbreviations: *D. glomerata*, D; *F. rubra*, F; *L. perenne*, L.

To identify bacterial taxa responsible for the observed differences among the three grass species, we performed an indicator species analysis to identify genera significantly associated with one, two, or all plant species. A total of 102 bacterial groups, comprising 33.4% of all bacterial taxa included in the analysis, were not significantly different in relative abundance and frequency with respect to grass species (Fig. 3, Supplementary Material Table S4). These groups belonged to three bacterial phyla, i.e., *Proteobacteria, Firmicutes*, and *Bacteroidetes*. Fifty-eight (19%) or 145 of all bacterial taxa (47.5%) were associated with one or two grass species, respectively (Fig. 3). Most unique bacterial taxa (9.2%) were associated with *L. perenne.* One uncultured *Rickettsiales* bacterium was significantly associated with *D. glomerata* while the two genera *Taibaiella* and *Luteimonas* as well as one uncultured *Sphingobacteriaceae* bacterium were significantly associated with *F. rubra*. In addition, twelve taxa including *Neorhizobium* and *Tumebacillus* were significantly associated with *L. perenne*.

**Figure 4:**
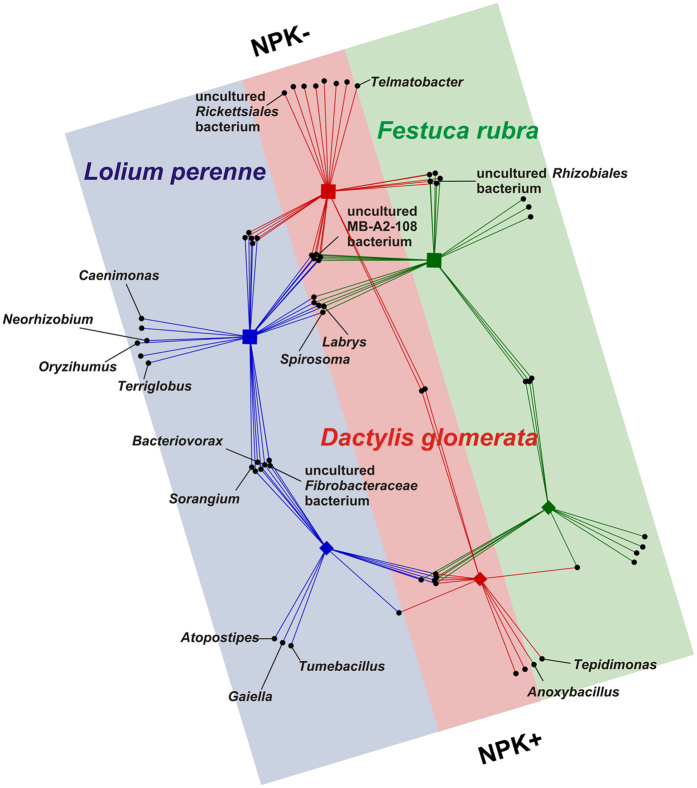
Bipartite association network of bacterial taxa with different treatments. Only significantly associated taxa are shown. Abbreviations: no fertilizer application, NPK-; with fertilizer application, NPK+.

As fertilizer application had a strong significant effect on bacterial community structures in all three grass species, an indicator analysis based on the two fertilization regimes was carried out. Twelve of the 305 bacterial taxa considered in this analysis showed a significant association with one of the three grass species with respect to the two fertilization regimes (Fig. 4, Supplementary Material Table S5). *Tepidimonas* and *Anoxybacillus* showed unique associations with *D. glomerata* grown on fertilized plots whereas *Telmatobacter* and a *Rickettsiales* member were significantly associated with *D. glomerata* plants grown on non-fertilized plots. The three taxa *Tumebacillus, Gaiella*, and *Atopostipes* showed unique associations with *L. perenne* grown on fertilized plots. In addition, four bacterial taxa (*Bacteriovorax, Neorhizobium, Oryzihumus*, and *Caenimonas*) were significantly associated with *L. perenne* grown on non-fertilized plots.

### Functional profiles of endophytic communities are altered by grass species and management

We further hypothesized that bacterial functioning is altered in a similar manner as bacterial endophyte diversity and community composition. To clarify this hypothesis, functional profiles were predicted from 16S rRNA gene data using the novel bioinformatic tool Taxa4Fun^17^ (Supplementary Material Table S6). Predicted functional profiles differed between the host plant species in 2011 but not in 2010 (Table 3). In addition, the combination of plant species with fertilizer application and/or mowing had a significant influence on predicted bacterial functioning in 2011. The combined effect of grass species, fertilizer application, and mowing frequency explained approximately 43% of the variance in the dataset. No significant impact of applied management regimes on bacterial community functions was detected for *D. glomerata* but for *L. perenne* in 2011 (Table 3). In addition, the putative functioning of bacterial endophytes in *F. rubra* in 2011 was marginally influenced by mowing frequency and the combination of fertilizer application with mowing frequency. Thus, plant species and management regimes affected not only diversity and structure but also the functional potential of the endophyte communities.

**Figure 5:**
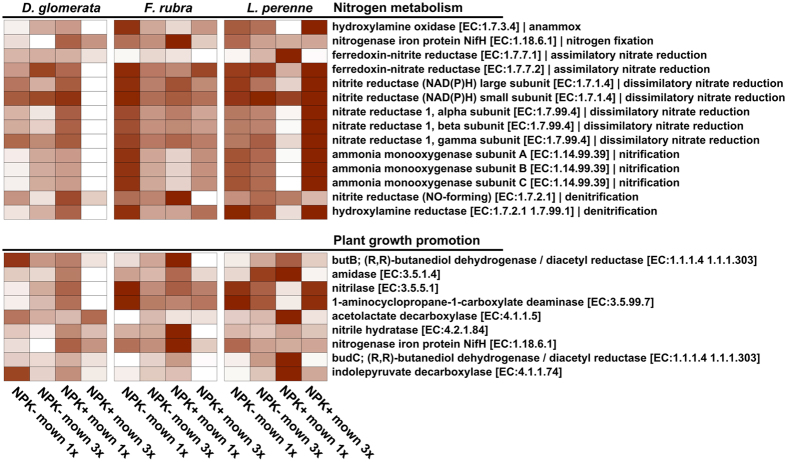
Predicted abundances of enzyme-encoding genes involved in plant growth promotion and nitrogen cycling. The color code refers to gene abundance, with high predicted abundances (red) and low predicted abundances (white). Abbreviations: *D. glomerata*, D; *F. rubra*, F; *L. perenne*, L.; no fertilizer application, NPK-; with fertilizer application, NPK+; mown three times a year, 3x; mown once a year, 1x.

To gain deeper insights into bacterial functioning, we focused on predicted abundances of genes involved in plant growth promotion, i.e., nitrilase, amidases, and nitrogenases, and on genes involved in the nitrogen cycle, such as nitrate reductase. Predicted gene abundances differed among the three grass species and the different management regimes (Fig. 5). In general, most genes were less abundant in *D. glomerata* compared to the other two grass species. Genes involved in nitrogen metabolism such as nitrate reductase [EC: 1.7.1.4] were predicted in higher or lower abundances in *D. glomerata* samples taken on fertilized plots mown once per year or on fertilized plots mown three times per year, respectively. In addition, higher average predicted abundances of genes involved in the nitrogen metabolism were observed in *L. perenne* and *F. rubra* plants collected from fertilized plots mown three times a year or from non-fertilized plots mown once a year, respectively. Such a clear pattern was not detected for genes potentially involved in plant growth promotion. For example, the predicted abundances of genes encoding nitrilases [EC: 3.5.1.4] and aminocyclopropane-1-carboxylate (ACC) deaminases [EC: 3.5.99.7] were lower in *D. glomerata* samples taken on plots with the lowest and highest management intensity while the opposite was observed for *L. perenne* samples. However, the highest predicted abundance of genes encoding amidases [EC: 3.5.1.4] was found in samples of all three grass species taken on fertilized plots mown once per year.

## Discussion

During the last years, several studies analyzed the response of bacterial endophytes towards management regimes such as fertilizer application. However, these studies mainly focused on root endophytes in a single plant species and/or on one management regime. To date, comparative studies on endophyte diversity and function in agriculturally important grass species are missing. This study is the first to apply pyrotag sequencing on the aerial bacterial endophyte community in important agricultural grass species. In total, 71 samples of the grass species *L. perenne, D. glomerata*, and *F. rubra* in two consecutive years were analyzed. Consistent with previous work^7^,^20^,^21^, bacterial endophyte communities were dominated by five phyla. The genera *Massilia, Pseudomonas*, and *Limnohabitans* were predominant in the three grass species. This is only partly in line with a previous study showing that *Pseudomonas, Rhizobium*, and *Bacillus* were the most abundant genera in roots and shoots of sorghum^21^. Similar results were obtained by Robinson *et al*.^7^ who found that *Pseudomonas* and *Bacillus* dominated the endophyte community in wheat roots and leaves, respectively.

Several of the abundant genera observed including *Bacillus, Rhizobium*, and *Stenotrophomonas* differed in their abundance with respect to management intensity. Members of the genera *Bacillus, Stenotrophomonas, Pseudomonas, Rhizobium*, and *Burkholderia* are well-known for their plant growth-promoting functions and/or the production of secondary metabolites including antibiotics or antifungal compounds^5^,^6^,^22^. In addition, genera including *Bacillus, Burkholderia, Pseudomonas*, and *Rhizobium* are reported as the most significant phosphate-solubilizing bacteria^22^. Moreover, several isolates of *Massilia* and *Luteimonas* were able to reduce nitrate^23^,^24^ suggesting an important role of these genera in the nitrogen cycle and thus as plant growth promoting bacteria. Nonetheless, we can only speculate that observed members of the above-mentioned genera have plant growth promoting abilities as strains of the same genus might differ in their plant growth promoting traits. In a previous study, the majority of plant-associated *Sphingomonas* spp. showed a protective effect against the plant pathogen *P. syringae,* although no general trait conserved within this genus was observed^25^.

Additionally, we detected genera containing well-known human and plant pathogens but obtained sequences were predominantly affiliated to uncultured bacteria within these genera. Members of the genus *Ralstonia* were observed as endophyte in several plant species such as red leaf and Iceberg lettuce^26^ or chili pepper^27^. In another study on the effect of pest management on bacterial endophyte communities in two grapevine cultivars (Merlot and Chardonnay), *Staphylococcus* was found in high numbers in Merlot plants^28^. *Ralstonia* was the dominant genus in both cultivars. The authors suggest that the prevalence of *Ralstonia* is linked to sampling of plants at the end of their vegetative cycle, which may enrich them in more saprophytic microbiota. This might explain our observation as we collected the grass species in autumn.

Grass species significantly affected bacterial diversity and community composition. This is supported by recent studies^29^,^30^,^31^,^32^ investigating endophyte communities in different plant species. McInroy and Kloepper^32^ showed that indigenous bacterial endophytes differed between cotton and sweet corn. However, this is the first study investigating endophyte communities in different agriculturally important grass species using a culture-independent, amplicon-based approach. No direct influence of fertilization and mowing frequencies on bacterial richness and diversity was observed. This observation is in contrast to two previous studies showing that fertilization influenced diversity and richness of diazotrophic bacteria^8^,^12^ but these bacteria comprise only a fraction of the entire endophyte community, which might account for the differences.

In the current study, fertilizer application significantly affected bacterial endophyte community composition. This is in line with previous research analyzing the effect of fertilizer application on endophytic diazotrophic bacteria in rice^10^,^12^. We recorded grass species-specific effects of management regimes on endophyte community composition. We suggest that these differences are related to differences in plant physiology. As mentioned above, *D. glomerata* and *L. perenne* exhibit a higher tolerance to mowing than *F. rubra* while *L. perenne* has a higher indicator value for nitrogen compared to the other two grass species^15^. It has been previously assumed that several factors including temperature, precipitation, or fertilizer application affect plant physiology and thus endophyte communtities^13^,^14^,^33^,^34^. As the combined effect of grass species, fertilizer application, and mowing frequency explained less than 50% of the variance in the dataset, we suggest that bacterial endophyte communities are affected by other factors such as sampling time or prevailing environmental conditions^7^,^33^,^35^. This is supported by differences in temperature and precipitation recorded for September 2010 and 2011.

Our correlation-based indicator species analysis indicates that the endophytes found in the three grass species form a core endophyte microbiota, which is conserved across different grass species. This is in accordance to Zinniel *et al*.^36^ who showed that different agronomic crop and prairie plant species harbor a significant set of indigenous bacterial endophytes mostly lacking a strict specificity. Our findings might be attributed to the different lifestyle strategies of endophytic species, which have been described previously^1^,^37^. Some bacteria are obligate endophytes and thus restricted to a life inside of plant tissues^37^. These obligate endophytes might constitute larger parts of the core community of the three grasses. Nonetheless, it has to be stated that this observation might also be attributed to active and passive bacterial colonization by competent and passenger endophytes, respectively^37^.

In addition, the results of correlation-based indicator species analysis support the assumption that different plant species select for (competent) endophytes as a result of advantages provided by these microbes^37^,^38^. This plant species-specific selection process results in different bacterial endophytic communities even between plant species growing next to each other. Many of the bacterial taxa observed such as *Caenimonas, Oryzihumus*, or *Tumebacillus* are common members of the soil microbiome^39^,^40^,^41^ while members of the genera *Geobacter, Schlegelella, and Planomicrobium* were previously described as endophytes^1^,^42^,^43^. However, the role of most microorganisms in the plant endosphere and their functions remain still unknown^1^,^34^.

We predicted functional profiles from 16S rRNA gene data using the novel bioinformatic tool Tax4Fun^19^ to study changes in bacterial functioning. Tax4Fun transforms the SILVA-based OTU classification into a taxonomic profile of KEGG organisms, which is subsequently normalized by the 16S rRNA copy number (obtained from NCBI genome annotations). Afterwards, KEGG profiles are converted into artificial metagenomes by combining functional profiles calculated for each of the KEGG genomes. These predicted metagenomes have been shown to be highly correlated with functional profiles derived from whole metagenome sequencing. Even for soils, which harbor several yet unknown or uncultured organisms, a Spearman correlation coefficient of 0.871 between functional profiles derived from the Tax4Fun prediction and direct metagenome sequencing approaches was obtained^19^. This indicates that Tax4Fun provides a good approximation to functional profiles obtained from metagenomic shotgun sequencing approaches. This is especially valuable to deduce functional profiles for a large number of samples derived from complex environments, as achieving a representative coverage for each sample of a large sample set would be extremely challenging.

Predicted functional profiles differed with regard to grass species in 2011 while mowing frequencies and fertilizer application only indirectly affected functional profiles as they might influence the grass species and thus bacterial functioning. These results indicate that the effect of management intensity on bacterial functions is plant species-dependent. We showed further that genes involved in plant growth promotion and in the nitrogen metabolism differed in their predicted abundances between the three grass species and the four management intensity levels. The ACC deaminase is involved in stress alleviation in plants (reviewed in ref. 1). ACC is a precursor of ethylene, which is a key regulator of the bacterial colonization of plant tissue^37^ and inhibits the nodule formation in legumes^44^. Nitrilases and amidases have been reported to play an important role in plant hormone production^1^. Nitrilases are also involved in the utilization of nitrogen compounds and in detoxification (reviewed in ref. 45). Because they reduce atmospheric nitrogen to ammonia, nitrogenases are key enzymes in nitrogen fixation, which is an efficient source of nitrogen for agriculture^46^. Nonetheless, it remains unclear why genes encoding these enzymes were less abundant in endophyte communities of *D. glomerata* or more abundant in *L. perenne* grown on with high intensity managed plots, respectively.

Fertilizer application altered the endophyte community composition in 2011 but not the functioning. In addition, mowing frequency had a significant effect on predicted bacterial functions but not on endophyte community composition, richness, and diversity in 2011. These differences might be explained by the fact that function and phylogeny of different bacteria are not necessarily related to each other. Vandenkoornhuyse *et al*.^38^ suggested that the core microbiome is functionally significant for the host plant. This supports the results of a previous study on bacterial communities associated with the green macroalga *Ulva australis*^47^. Here, a high phylogenetic variability in bacterial species composition and a high similarity of the functional composition was observed, indicating an existing functional redundancy. In another study on root-associated bacterial communities, plant–host-species selectivity was more closely related to specific metabolic activities, such as polysaccharide degradation and anaerobic respiration^48^. In the current study, we did not observe any difference in community function but composition in 2010 also supporting the idea of functional redundancy of different community members between the investigated grass species. However, the opposite was recorded in 2011. This observation might be related to functional differences conferred by an ‘accessory’ microbiome unique for each plant^38^. As consequence, further studies are needed to better understand how management regimes affect functional traits of bacterial endophyte communities.

## Conclusion

In the present study, we showed that grass species had a significant effect on bacterial endophyte diversity and community composition. These results are in line with our first hypothesis that bacterial endophyte diversity and community composition differed between the grass species. We further demonstrated that observed management effects on bacterial endophytes were grass species-dependent which supports our second hypothesis. These results suggest that grass species rather than grassland management regimes are the key driver of bacterial endophyte diversity and community composition. Functional analysis revealed that the predicted abundance of bacterial genes involved in plant growth promotion and the nitrogen metabolism differed between the three grass species and the management intensity levels. In contrast to our third hypothesis, bacterial functioning was affected in a different manner as bacterial endophyte community composition and diversity indicating that bacterial endophyte community composition is not necessarily linked with bacterial functioning. Nonetheless, the results of the present work along with the application of a novel bioinformatic approach resulted in a holistic picture of compositional and functional responses of bacterial endophytes in agriculturally relevant grass species towards management practices.

## Materials and Methods

### Sampling

Sampling was performed as described previously^9^. In brief, aerial plant parts of *L. perenne* L., *F. rubra* L., and *D. glomerata* L. were collected on 19^th^ September 2010 and 12^th^ September 2011 from the Grassland Management Experiment (GrassMan). The experimental design included four different management intensity levels: (1) no fertilizer application and mown once a year, (2) no fertilizer application and mown thrice per year, (3) fertilizer application with nitrogen/phosphorous/potassium (NPK) and mown once a year, and (4) fertilizer application with NPK and mown thrice a year (Supplementary Material Fig. S1). For a detailed description of the design and the management regimes, see Wemheuer *et al*.^9^ and Petersen *et al*.^17^. Three samples per treatment and grass species were taken in both sampling years with one exception: *L. perenne* samples were collected only on two non-fertilized plots mown once a year in 2010 due to the absence of this plant species on these plots. In total, 71 plant samples were analyzed in this study (Supplementary Material Table S1). One sample comprised ten individual plants. Only plants without obvious disease symptoms such as leaf spots, chlorosis, or pathogen-induced lesions were collected. Plant samples were immediately cooled to 4 °C and transferred to the laboratory. During the study period, precipitation and mean temperature were 93.6 mm and 11.42 °C in September 2010 and 54.75 mm and 14.75 °C in September 2011, respectively.

### Surface sterilization and extraction of total community DNA

Surface sterilization of collected plant material was performed as described in Wemheuer *et al*.^9^. To confirm the success of the surface sterilization, 100 μl aliquots of the water used in the final washing step were plated on common laboratory media plates. The plates were incubated in the dark at 25 °C for at least 2 weeks. No growth of microorganisms was observed. In addition, water from the final washing step was subjected to PCR targeting the 16S rRNA gene. No amplification of 16S rRNA gene was detected. The surface-sterilized plant material was ground to a fine powder in liquid nitrogen using an autoclaved mortar and pestle. Ground tissue powder aliquots were subsequently stored at −20 °C until DNA extraction. Total microbial community DNA was extracted employing the peqGOLD Plant DNA Mini kit (Peqlab, Erlangen, Germany; now VWR) according to the manufacturer’s instructions with two modifications as described previously^9^. The modifications included a beat-beating step using glass beads and the addition of proteinase K.

### Amplification of 16S rRNA genes

Bacterial endophyte communities were assessed using a nested PCR approach targeting the 16S rRNA gene. For details of the first PCR reaction mixture and the thermal cycling scheme see Wemheuer *et al*.^9^. In brief, the primers 799 f (AACMGGATTAGATACCCKG)^49^ and 1492 R (GCYTACCTTGTTACGACTT)^50^ were used in the first PCR to suppress co-amplification of chloroplast-derived 16S rRNA genes^49^. PCR amplification resulted in two PCR products: a mitochondrial product with approximately 1.1 kbp and a bacterial product of approximately 735 bp. Genomic DNA of *Bacillus licheniformis* DSM13 was used as control for the bacterial product. Three independent PCRs were performed per sample. Bacteria-specific bands were purified using the peqGOLD Gel Extraction kit (Peqlab) according to the manufacturer’s instructions, quantified using a Nanodrop (ND-1000) (Peqlab) and subjected to the nested PCR reaction.

The V6-V8 region of the 16S rRNA gene was amplified with primers containing the Roche 454 pyrosequencing adaptors and key (both underlined) as well as one unique MID per sample: F968 5′-CCATCTCATCCCTGCGTGTCTCCGAC-TCAG-(dN)_16_- AACGCGAAGAACCTTAC-3′ and R1401 5′-CCTATCCCCTGTGTGCCTTGGCAGTC-TCAG-CGGTGTGTACAAGACCC-3′^51^. The PCR reaction (25 μl) contained 5 μl of five-fold Phusion HF buffer, 200 μM of each of the four deoxynucleoside triphosphates, 4 μM of each primer, 2 U of Phusion high fidelity hot start DNA polymerase (Thermo Scientific, Waltham, MA, USA) and approximately 10 ng of the first PCR product as template. We used the same template in the second PCR as in our previous DGGE-based study^9^. The following thermal cycling scheme was used: initial denaturation at 98 °C for 30 s, 30 cycles of denaturation at 98 °C for 15 s, annealing at 53 °C for 30 s, followed by extension at 72 °C for 30 s. The final extension was carried out at 72 °C for 2 min. Negative controls were performed using the reaction mixture without template. Obtained PCR products were controlled for appropriate size and subsequently purified using the peqGOLD Gel Extraction kit (Peqlab) as recommended by the manufacturer. PCR products were quantified using Quant-iT dsDNA HS assay kit and a Qubit fluorometer (Thermo Scientific) as recommended by the manufacturer. Purified PCR products from the three independent PCRs were subsequently pooled in equal amounts. The Göttingen Genomics Laboratory determined the 16S rRNA gene sequences employing the Roche GS-FLX + pyrosequencer with Titanium chemistry (Roche, Mannheim, Germany).

### Processing of pyrosequencing-derived datasets

Obtained sequences were demultiplexted and quality filtered employing the QIIME 1.8.0 software package^52^. During this step, short sequences (<250 bp,) with long homopolymer stretches (>8 bases) and primer mismatches (>3 bases) were removed. Afterwards, sequences were denoised with Acacia version 1.53b^53^. Remaining reverse primer sequences were truncated employing cutadapt version 1.0^54^. Chimeric sequences were removed with Usearch version 7.0.190^55^ in *denovo* and in reference mode using the Silva SSURef 119 NR database^56^. All processed sequences of each sample were concatenated into a single file and subsequently clustered *denovo* in operational taxonomic units (OTUs) at 97% genetic identity as described previously^57^. A consensus sequence for each OTU was classified by BLAST alignment^58^ against the Silva SSURef 119 NR database^56^ using QIIME to determine taxonomy. Only OTUs of bacterial origin were considered for further analysis. In addition, OTUs occurring in less than three samples were removed. The final OTU table is provided as Supplementary Material Table S2. Rarefaction curves and alpha diversity indices (Richness and Shannon) were calculated using R version 3.3.1^59^ and the vegan package^60^ (Supplementary Material Table S3). In addition, the drc package^61^ was used in R to calculate the Michaelis-Menten Fit for sampling depth estimation (Supplementary Material Table S3). The OTU table was rarefied to 125 sequences per sample prior to calculation of alpha diversity and coverage. All diversity indices and richness estimators were calculated ten times. The average is provided for each sample. Functional profiles were predicted from the 16S rRNA data using Tax4Fun^19^ with short read mode disabled (Supplementary Material Table S6).

### Statistical analysis

All statistical analyses were performed in R version 3.3.1^59^. The impact of grass species and management regimes on bacterial community structure and function was evaluated by PERMANOVA using Bray-Curtis and weighted UniFrac distance matrices. We also tested unweighted and generalized UniFrac distances. However, these displayed a lower environmental sensitivity based on the lower coefficients of determination. Distance matrices were generated in R using the GUniFrac package^62^. The OTU table used for beta diversity calculation were rarefied to 125 sequences per sample prior to beta diversity analysis. The rarefied OTU table is provided as Supplementary Material Table S7. In addition, count data were transformed to proportional data which were subsequently used for calculation (Supplementary Material Table S8). Obtained results were highly similar compared to those from the rarefied OTU table with one exception. The combined effect of fertilizer application and mowing frequency on endophyte communities of *L. perenne* in 2010 was only significant when using the rarefied table although a marginally significant influence was observed. The neighbor-joining tree used for UniFrac analyses was calculated from OTU consensus sequences using muscle version 3.8.31^63^. The homogeneity of the different replicates with respect to treatment and grass species were visualized by nonmetric-multidimensional scaling indicating a high similarity between the bacterial community structures in the three replicate samples (Supplementary Material Fig. S3). Differences in richness and diversity with respect to grass species or management regime were tested by Kruskal-Wallis test and subsequently by pairwise t test with Bonferroni-corrected P values. Changes were considered significant with P ≤ 0.05. Samples taken in 2010 and 2011 were analyzed separately to avoid temporal pseudoreplication.

To identify the bacterial assemblages associated with the different grass species, correlation-based indicator species analysis was performed using *multipatt* (indicSpecies)^18^. The abundances of all OTUs affiliated to the same genus were summarized prior to analysis. For visualization, a bipartite network was generated using the three grass species per treatment, i.e., each grass species as either fertilized or non-fertilized, as source nodes, and the genera as target nodes. All taxa with a possible association were visualized but only those with significant (P ≤ 0.05) associations were identified in the networks. Network generation was performed using the *edge-weighted spring embedded layout* algorithm in *Cytoscape*^64^, with the edge weight corresponding to the association strength of each genus with each treatment. The results of the indicator analyses with regard to grass species and to grass species combined with fertilizer application are provided as Supplementary Material Tables S4 and S5, respectively.

### Nucleotide sequence accession numbers

Sequence data are deposited in the Sequence Read Archive (SRA) of the National Center for Biotechnology Information (NCBI) under the accession number SRA419370.

## Additional Information

**How to cite this article**: Wemheuer, F. *et al*. Bacterial endophyte communities of three agricultural important grass species differ in their response towards management regimes. *Sci. Rep.* **7**, 40914; doi: 10.1038/srep40914 (2017).

**Publisher's note:** Springer Nature remains neutral with regard to jurisdictional claims in published maps and institutional affiliations.

## Supplementary Material

Supplementary Information

Supplementary Table S1

Supplementary Table S2

Supplementary Table S3

Supplementary Table S4

Supplementary Table S5

Supplementary Table S6

Supplementary Table S7

Supplementary Table S8

## Figures and Tables

**Table 1:** Impact of grass species and management regimes on richness and diversity of bacterial endophyte communities.

	Richness		Diversity	
	2010	2011	2010	2011
***All grass species***				
Grass species	0.617	0.508	0.002	0.006
Fertilization	*0.062*	0.146	0.179	0.206
Mowing	0.934	1.000	0.812	0.825
Grass species + Fertilization	0.161	0.149	0.012	0.034
Grass species + Mowing	0.844	0.839	0.010	0.027
Fertilization + Mowing	0.208	0.428	0.560	0.605
Grass species + Fertilization + Mowing	*0.068*	*0.059*	*0.063*	0.124
***Dactylis glomerata***				
Fertilization	*0.055*	*0.055*	0.109	*0.078*
Mowing	0.873	0.873	0.873	0.749
Fertilization + Mowing	0.033	*0.060*	0.442	0.248
***Festuca rubra***				
Fertilization	0.631	0.522	0.423	0.873
Mowing	0.873	0.873	0.262	0.262
Fertilization + Mowing	*0.053*	*0.072*	0.546	0.727
***Lolium perenne***				
Fertilization	0.410	0.361	0.810	1.000
Mowing	0.522	0.361	0.109	0.150
Fertilization + Mowing	0.680	0.433	0.110	0.294

Significant (P ≤ 0.05) and marginally significant (P ≤ 0.10) P values are underlined and written in italics, respectively.

**Table 2:** Impact of grass species and management regimes on structure of bacterial endophyte communities. Distances were calculated using a rarefied OTU table.

Distance measure	Bray-Curtis				Weighted UniFrac			
Year	2010		2011		2010		2011	
	R^2^	P	R^2^	P	R^2^	P	R^2^	P
**All grass species**								
Grass species	12.92%	0.002	12.10%	0.003	17.88%	0.002	10.11%	0.014
Fertilization	4.32%	0.106	*4.36%*	*0.064*	3.88%	0.179	3.86%	0.177
Mowing	1.63%	0.992	3.03%	0.358	1.64%	0.921	2.87%	0.43
Grass + Fertilization	23.48%	0.002	21.74%	0.004	27.84%	0.001	*19.17%*	*0.052*
Grass + Mowing	20.85%	0.006	19.48%	0.014	25.43%	0.001	18.94%	0.038
Fertilization + Mowing	9.25%	0.337	12.35%	0.024	8.56%	0.46	*11.42%*	*0.088*
Grass + Fertilization + Mowing	42.51%	0.002	41.21%	0.003	45.24%	0.005	43.04%	0.002
***Dactylis glomerata***								
Fertilization	15.54%	0.028	10.69%	0.209	19.91%	0.027	9.81%	0.397
Mowing	8.99%	0.414	11.29%	0.179	8.48%	0.418	10.01%	0.395
Fertilization + Mowing	37.65%	0.014	29.15%	0.319	41.13%	0.021	30.78%	0.242
***Festuca rubra***								
Fertilization	6.97%	0.537	10.94%	0.222	5.16%	0.892	7.90%	0.574
Mowing	5.66%	0.771	6.41%	0.83	5.76%	0.784	8.72%	0.459
Fertilization + Mowing	27.69%	0.332	*32.18%*	*0.088*	23.67%	0.719	31.58%	0.195
***Lolium perenne***								
Fertilization	11.99%	0.214	11.26%	0.192	7.44%	0.789	11.56%	0.213
Mowing	*12.55%*	*0.098*	7.33%	0.583	14.19%	0.147	10.30%	0.319
Fertilization + Mowing	34.55%	0.048	37.71%	0.103	31.57%	0.359	*44.47%*	*0.062*

Significant (P ≤ 0.05) and marginally significant (P ≤ 0.10) parameters are underlined and written in italics, respectively.

**Table 3:** Impact of grass species and management regimes on function of bacterial endophyte communities.

Year	2010		2011	
	R^2^	P	R^2^	P
**All grass species**				
Grass species	7.18%	0.228	11.13%	0.03
Fertilization	1.44%	0.885	2.06%	0.614
Mowing	1.71%	0.788	6.82%	0.041
Grass + Fertilization	13.1%	0.648	16.68%	0.245
Grass + Mowing	14.16%	0.487	24.69%	0.01
Fertilization + Mowing	5.36%	0.951	*13.37%*	*0.072*
Grass + Fertilization + Mowing	30.4%	0.642	43.46%	0.015
***Dactylis glomerata***				
Fertilization	6.69%	0.555	5.91%	0.666
Mowing	5.57%	0.72	14.9%	0.157
Fertilization + Mowing	25.22%	0.505	25.44%	0.475
***Festuca rubra***				
Fertilization	4.52%	0.908	5.55%	0.678
Mowing	5.76%	0.795	*19.93%*	*0.063*
Fertilization + Mowing	21%	0.815	*44.32%*	*0.057*
***Lolium perenne***				
Fertilization	6.69%	0.838	8.12%	0.491
Mowing	13.75%	0.141	11.06%	0.257
Fertilization + Mowing	26.92%	0.658	61.44%	0.017

Significant (P ≤ 0.05) and marginally significant (P ≤ 0.10) parameters are underlined and written in italics, respectively.
