# Supplementary material for: Bacterial endophyte communities of three agricultural important grass species differ in their response towards management regimes
Source: Sci Rep. 2017 Jan 19;7:40914. doi: 10.1038/srep40914 (PMC5244420; doi:10.1038/srep40914)
Supplement: Supplementary Information [file srep40914-s1.pdf]

For submission to Scientific Reports

Supplementary Information

Bacterial endophyte communities of three agricultural important grass species differ in their response towards management regimes

Franziska Wemheuer, Kristin Kaiser, Petr Karlovsky, Rolf Daniel, Stefan Vidal, Bernd Wemheuer

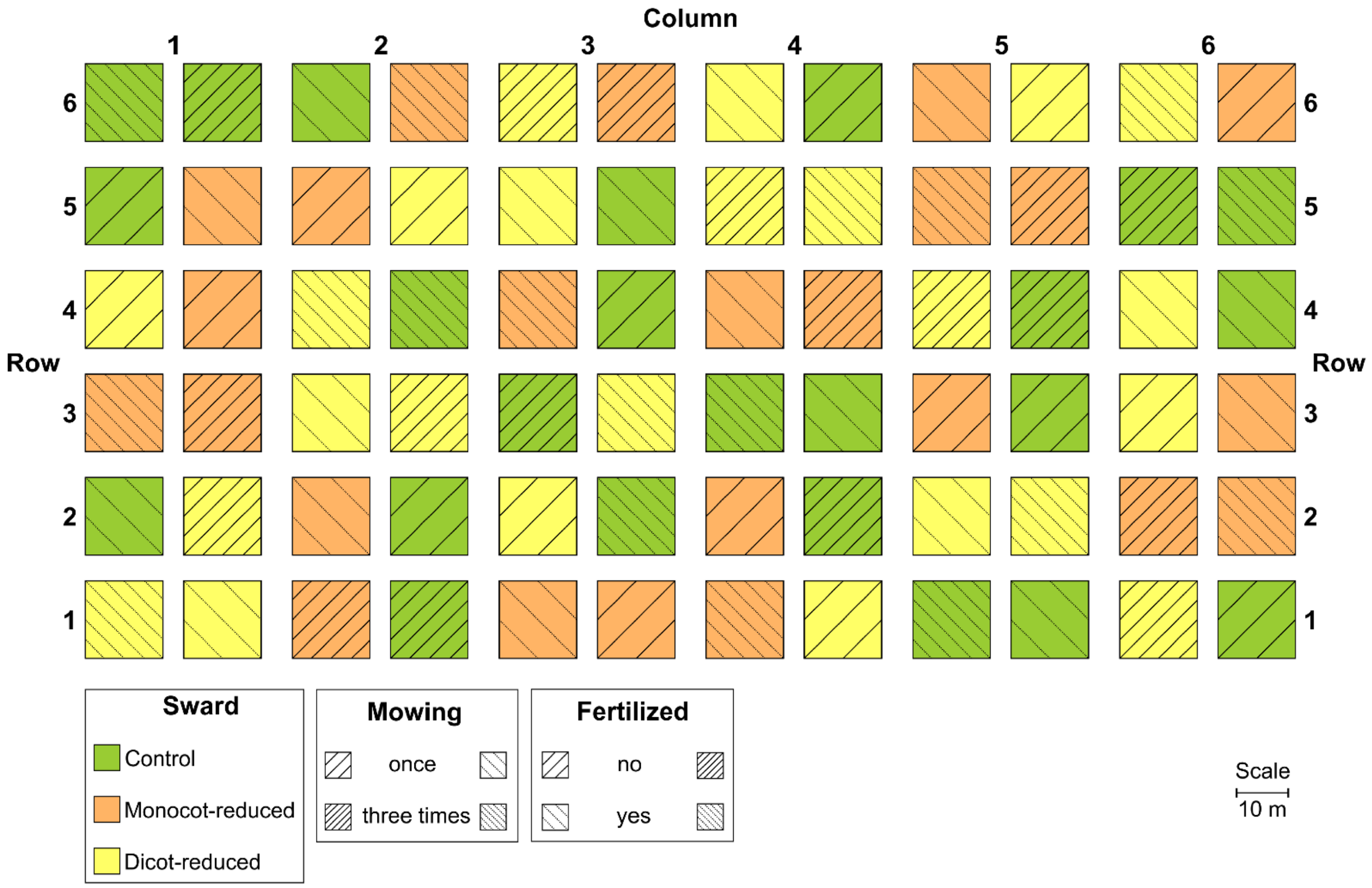

Figure S1. Experimental setup of the GrassMan experimental field.

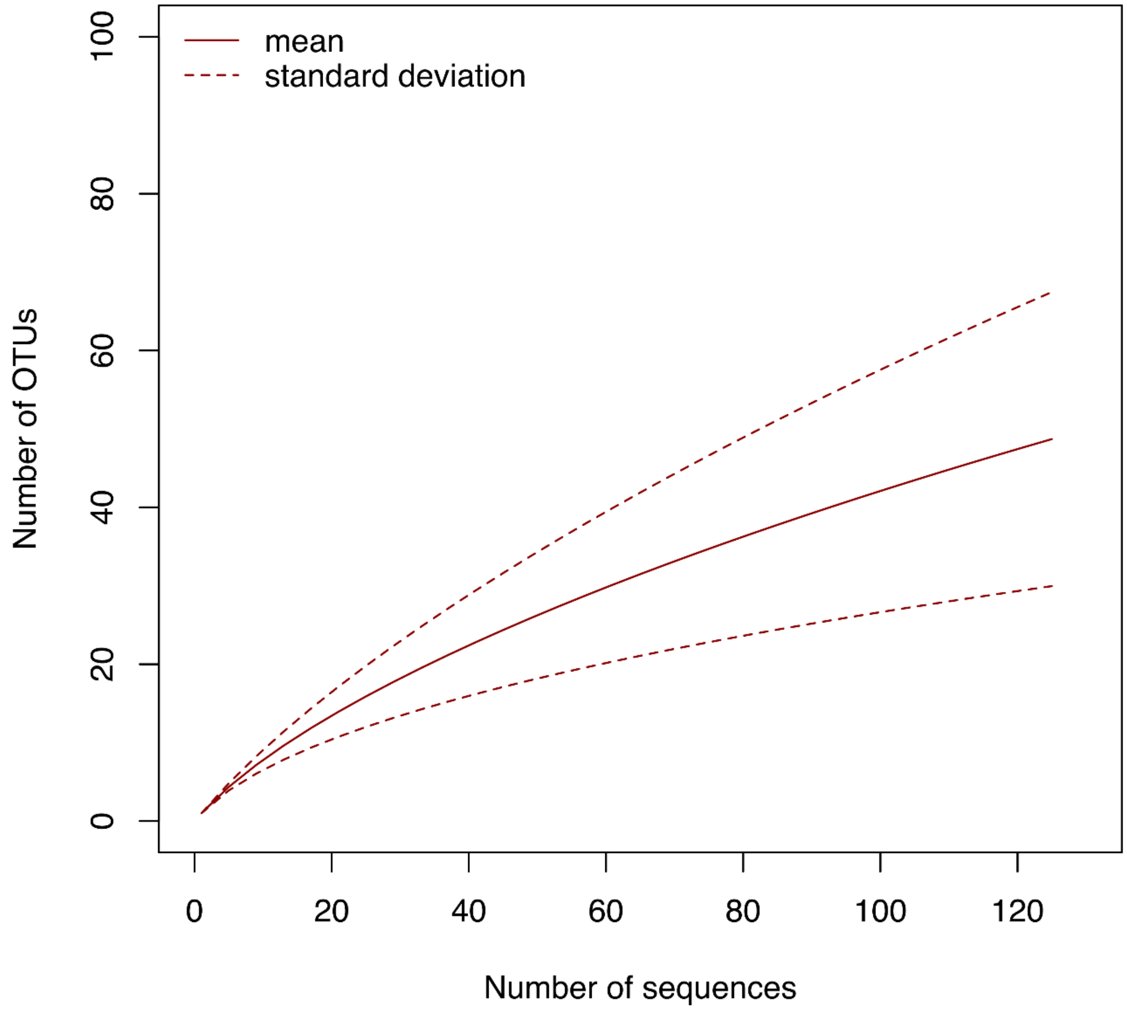

Figure S2. Rarefaction curves. Only the mean of all curves and the standard deviation are shown.

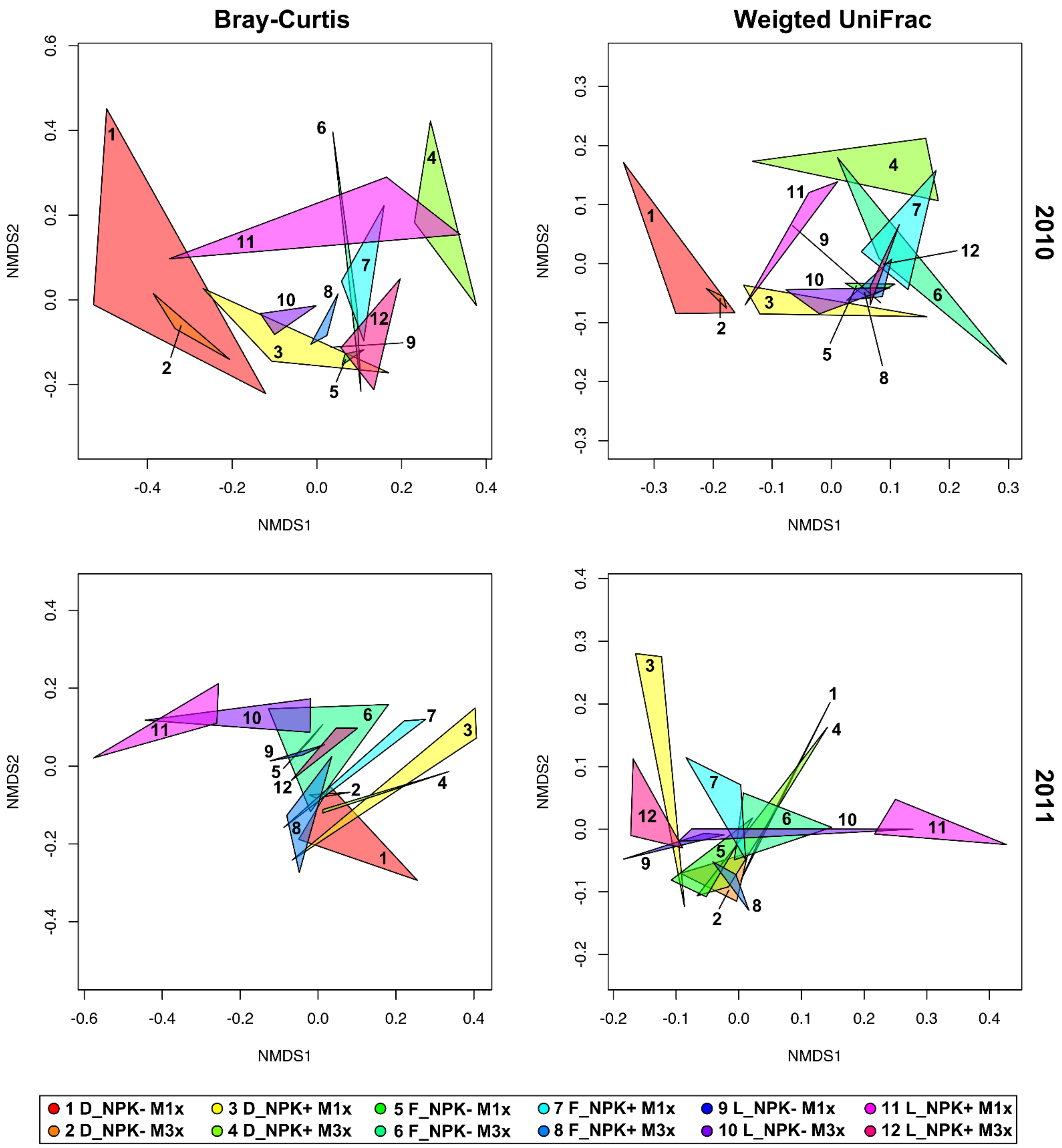

Figure S3. Observed variance for each set of replicates. Each triangle represents the 3 replicates of each treatment (1-12). Abbreviations: *D. glomerata*, D; *F. rubra*, F; *L. perenne*, L.; no fertilizer application, NPK-; with fertilizer application, NPK+; mown three times a year, M3x; mown once a year, M1x.

(Supplementary Tables are provided as extra files)

Table S1. Sampling Data.

Table S2. OTU table for the bacterial endophyte communities. OTUs were clustered at 3% genetic distance. For further information on the Sample ID see Table S1 in the supplemental material.

Table S3. Richness and diversity of the bacterial community calculated with the same surveying effort (n = 125). Richness is expressed as the number of OTUs. For further information on the Sample ID see Table S1 in the supplemental material.

Table S4. Indicator species analysis for the three grass species. Stat = association value.

Table S5. Indicator species analysis for the three grass species with respect to fertilization. Stat = association value.

Table S6. Functional profiles calculated for the bacterial endophyte communities. For further information on the Sample ID see Table S1 in the Supplemental Material.

Table S7. Rarefied OTU table used for beta-diversity analysis. Each sample was rarefied to 125 sequences. For further information on the Sample ID see Table S1 in the supplemental material.

Table S8. Impact of grass species and management regimes on structure of bacterial endophyte communities. Distances were calculated based on proportional data. Significant ($P \leq 0.05$) and marginally significant ($P \leq 0.10$) parameters are underlined and written in italics, respectively.
